# Early-Onset Epstein-Barr Virus (EBV)-Positive Post-transplant Lymphoproliferative Disorder Presenting With Catastrophic Gastrointestinal Hemorrhage and Ischemic Bowel Following Kidney Transplantation

**DOI:** 10.7759/cureus.91859

**Published:** 2025-09-08

**Authors:** Srikanth Davuluri, Majed Jandali, Tyler Paul, Kenneth Wind

**Affiliations:** 1 Pulmonary and Critical Care Medicine, Rosalind Franklin University of Medicine and Sciences, North Chicago, USA; 2 Pulmonary and Critical Care Medicine, Froedtert South Pleasant Prairie Hospital, Pleasant Prairie, USA; 3 Surgery, Froedtert South Pleasant Prairie Hospital, Pleasant Prairie, USA; 4 Internal Medicine, Rosalind Franklin University of Medicine and Sciences, North Chicago, USA; 5 Pathology, Froedtert South Pleasant Prairie Hospital, Pleasant Prairie, USA

**Keywords:** diffuse large b-cell lymphoma, ebv-associated lymphoma, epstein barr virus (ebv), gastro intestinal haemorrhage, kidney transplant, post transplant lymphoproliferative disorder (ptld), rituximab, small bowel ischemia

## Abstract

Post-transplant lymphoproliferative disorder (PTLD) is a rare but fatal outcome following solid organ transplant, which is often associated with Epstein-Barr virus (EBV) infection or immunosuppression. Subsequent gastrointestinal involvement is uncommon and can lead to severe complications.

We describe a 58-year-old male who developed EBV-associated PTLD within six months of a deceased donor renal transplant who presented with persistent severe gastrointestinal bleeding. Multiple surgical resections were required as the patient also developed progressive ischemic bowel disease. Despite immunosuppression reduction, rituximab therapy, and surgical intervention, his condition worsened and led to multi-organ failure and eventually death.

This case highlights the aggressive clinical course of gastrointestinal-associated PTLD and the challenges in diagnosis and management. Early recognition with multidisciplinary care is critical for treatment, but may be insufficient in cases with advanced disease. EBV monitoring and tailored immunosuppression in transplant recipients are also essential in improving outcomes in high-risk patients.

## Introduction

Post-transplant lymphoproliferative disorder (PTLD) is an uncommon but potentially life-threatening complication of solid organ transplantation, occurring in approximately 1-3% of kidney transplant recipients and more frequently in heart-lung recipients [1-3]. The majority of cases are associated with Epstein-Barr virus (EBV)-driven B-cell proliferation under chronic immunosuppression, most often with calcineurin inhibitors and antimetabolites [4-6]. Extranodal involvement is common, and while gastrointestinal (GI) manifestations occur in 10-15% of patients, clinically significant bleeding and ischemia are less frequent but associated with high morbidity and mortality. Bowel ischemia in PTLD may result from direct tumor infiltration, vascular compromise following angio-embolization, or hypoperfusion in the setting of massive hemorrhage and vasopressor use [7,8]. Despite advances in surveillance and therapeutic strategies, severe GI PTLD remains difficult to manage, and outcomes are often poor [9-11]. We present a case of early-onset EBV-positive PTLD with catastrophic GI bleeding and ischemic bowel following kidney transplantation, highlighting the diagnostic and therapeutic challenges in this setting.

## Case presentation

A 58-year-old male with end-stage renal disease secondary to IgA nephropathy underwent a deceased-donor kidney transplant in March 2024. His maintenance immunosuppressive regimen included tacrolimus and mycophenolate mofetil (MMF).

Six months post-transplant, in August 2024, the patient presented with weakness, anemia (hemoglobin 9.1 g/dL), and rectal bleeding. Baseline creatinine was 2.2-2.6 mg/dL. Admission vitals were weight 175 lb, height 180.3 cm, BMI 24.5, blood pressure 118/56 mmHg, heart rate 73 bpm, temperature 97.9 °F, and SpO₂ 98% on room air. Initial colonoscopy revealed large internal and moderate external hemorrhoids, which were managed conservatively with hydrocortisone cream and the patient was subsequently discharged from the hospital.

He was readmitted the following month in September 2024, with recurrent bright red blood per rectum, weakness, and anemia (hemoglobin 7.4 g/dL). Iron studies showed elevated ferritin with normal iron, folate, and vitamin B12. Despite multiple blood transfusions (total of four units packed red blood cells (PRBC)), bleeding persisted. On September 17, 2024, CT angiography demonstrated active jejunal bleeding, and arterial foam embolization was performed.

**Figure 1:**
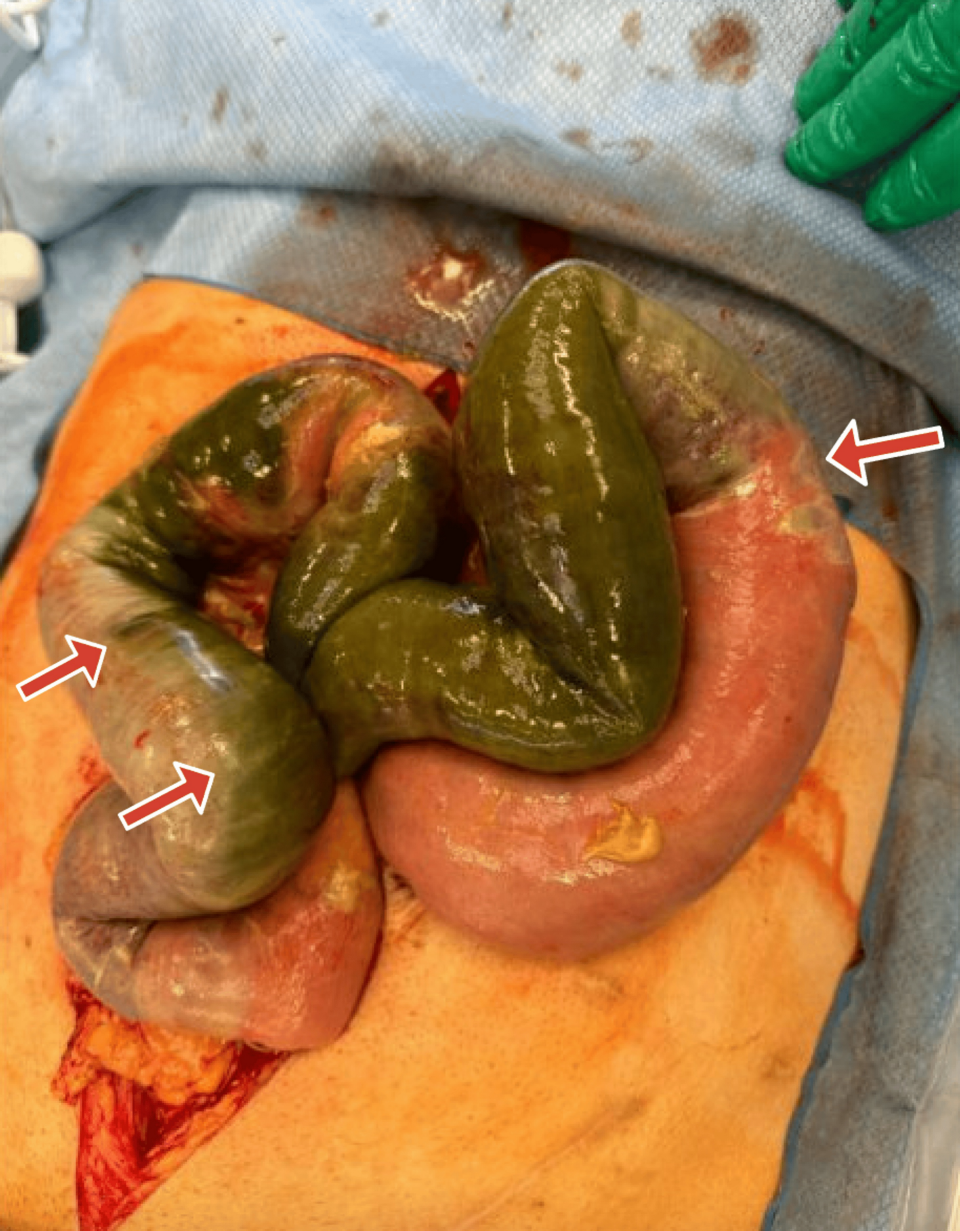
Postoperative diagnosis of ischemia of the proximal small bowel for about 30 to 40 cm of the jejunum.

Over the following days, he developed severe abdominal pain, prompting surgical consultation for suspected ischemia. An emergent laparotomy in September 2024 revealed 30-40 cm of ischemic proximal jejunum (Figure 1), which was resected with a side-to-side anastomosis. Bleeding subsided briefly but recurred, necessitating additional transfusions. Concurrently, the patient developed oliguria with worsening renal function, creatinine rose to 6.1 mg/dL with blood urea nitrogen (BUN) 183 mg/dL, requiring initiation of renal replacement therapy.

**Figure 2:**
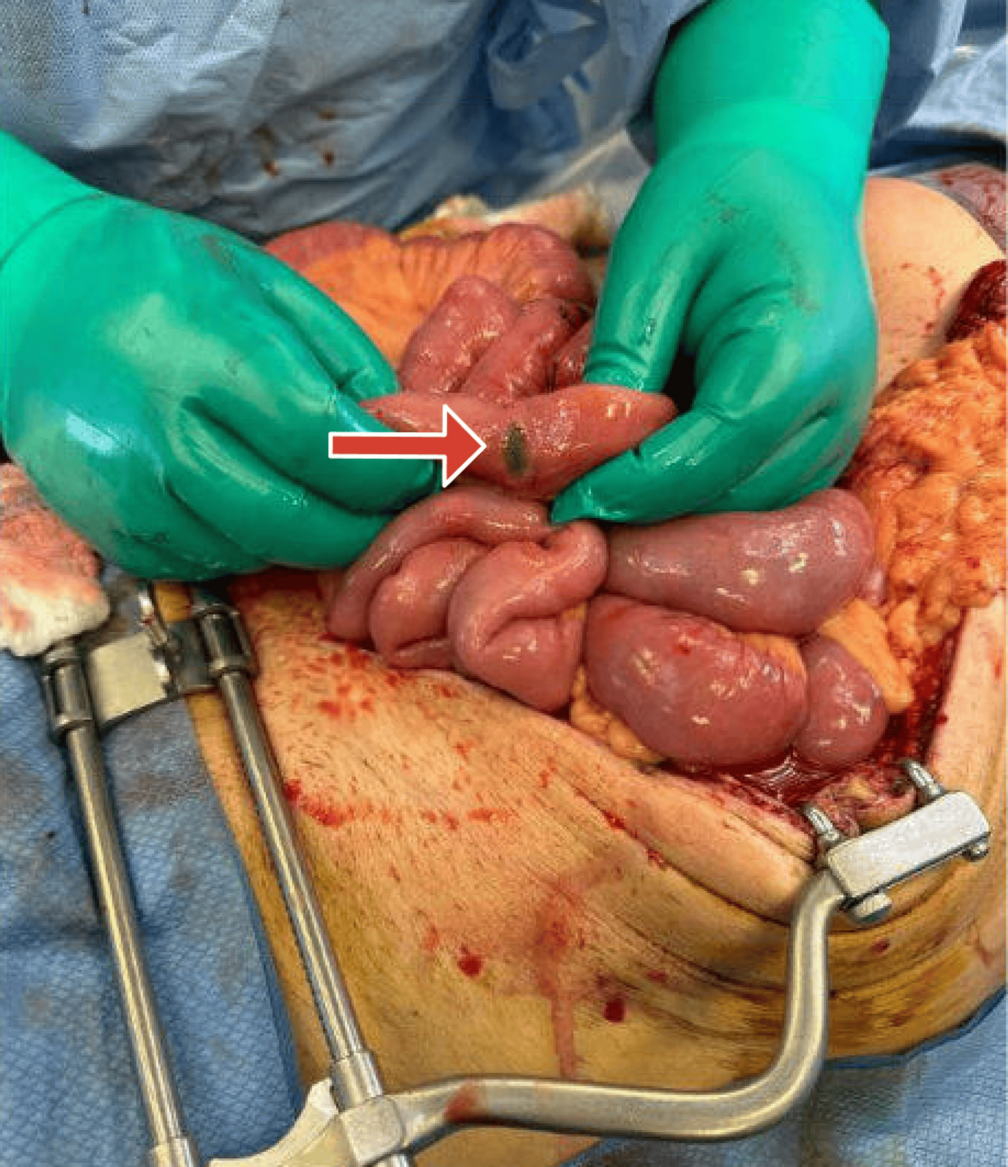
Multiple small area of obvious small bowel necrosis spread throughout the small bowel distal to the anastomosis.

**Figure 3:**
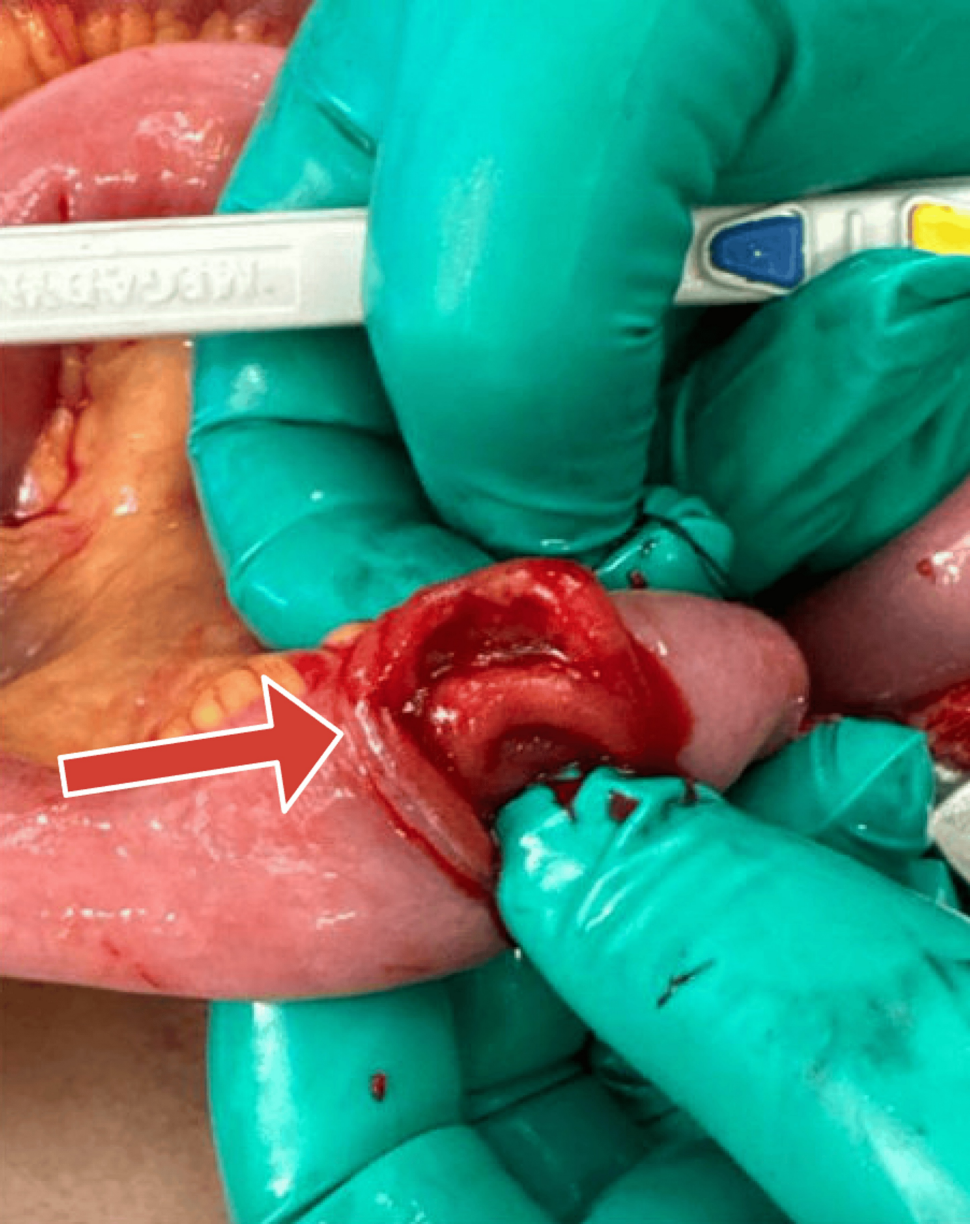
Additional small bowel showing ulceration.

**Figure 4:**
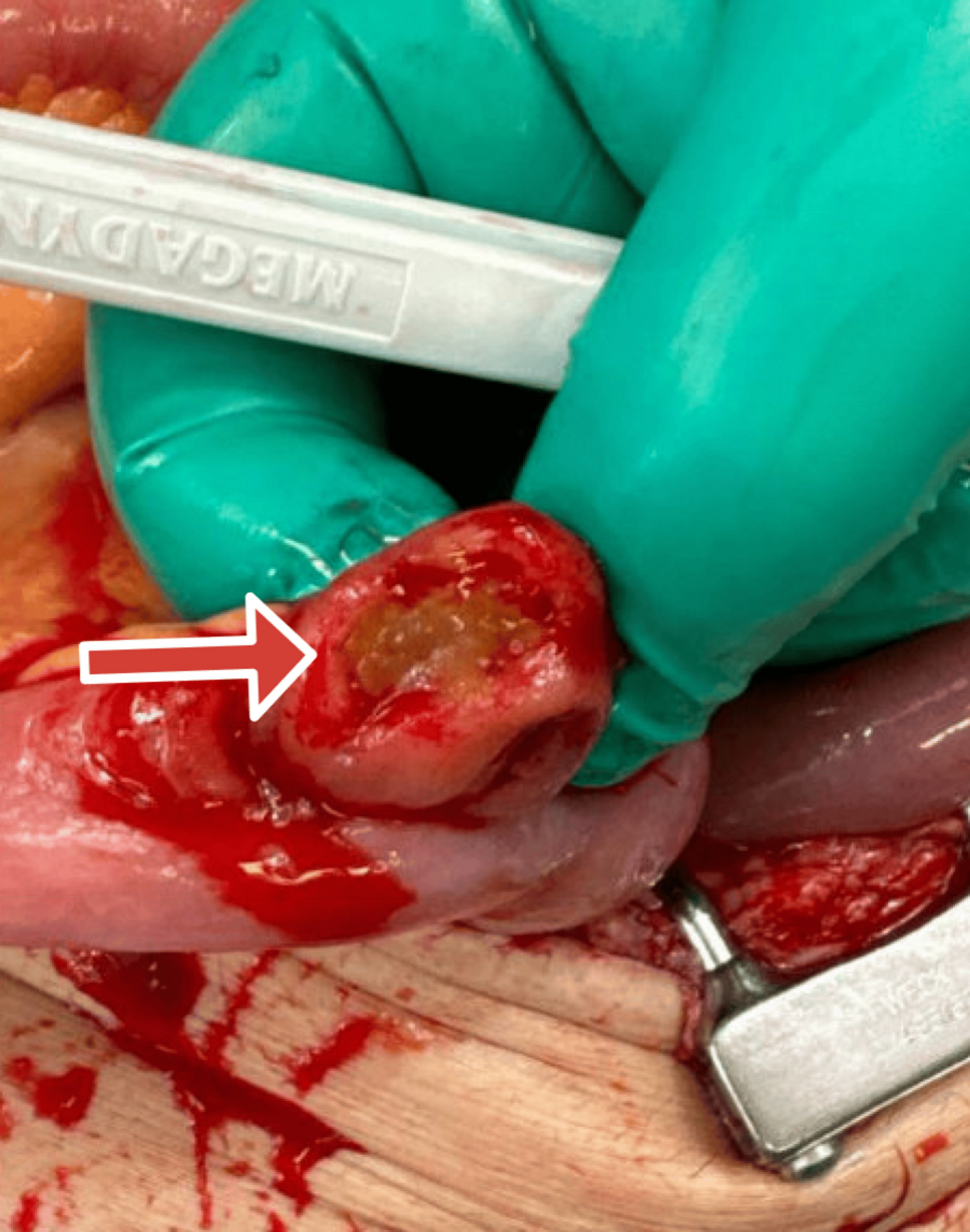
Additional close-up image of focal bowel necrosis.

Subsequently his hemoglobin dropped to 5.4 g/dL despite multiple transfusions. Continued hemorrhage with hemodynamic instability necessitated repeat laparotomy with push enteroscopy in October 2024. Findings included multiple ischemic ulcerations (Figures 2, 3) and diffuse small bowel involvement (Figure 4). Additional resections were performed, with jejunoileal anastomosis, leaving approximately 100 cm of viable small bowel.

**Figure 5:**
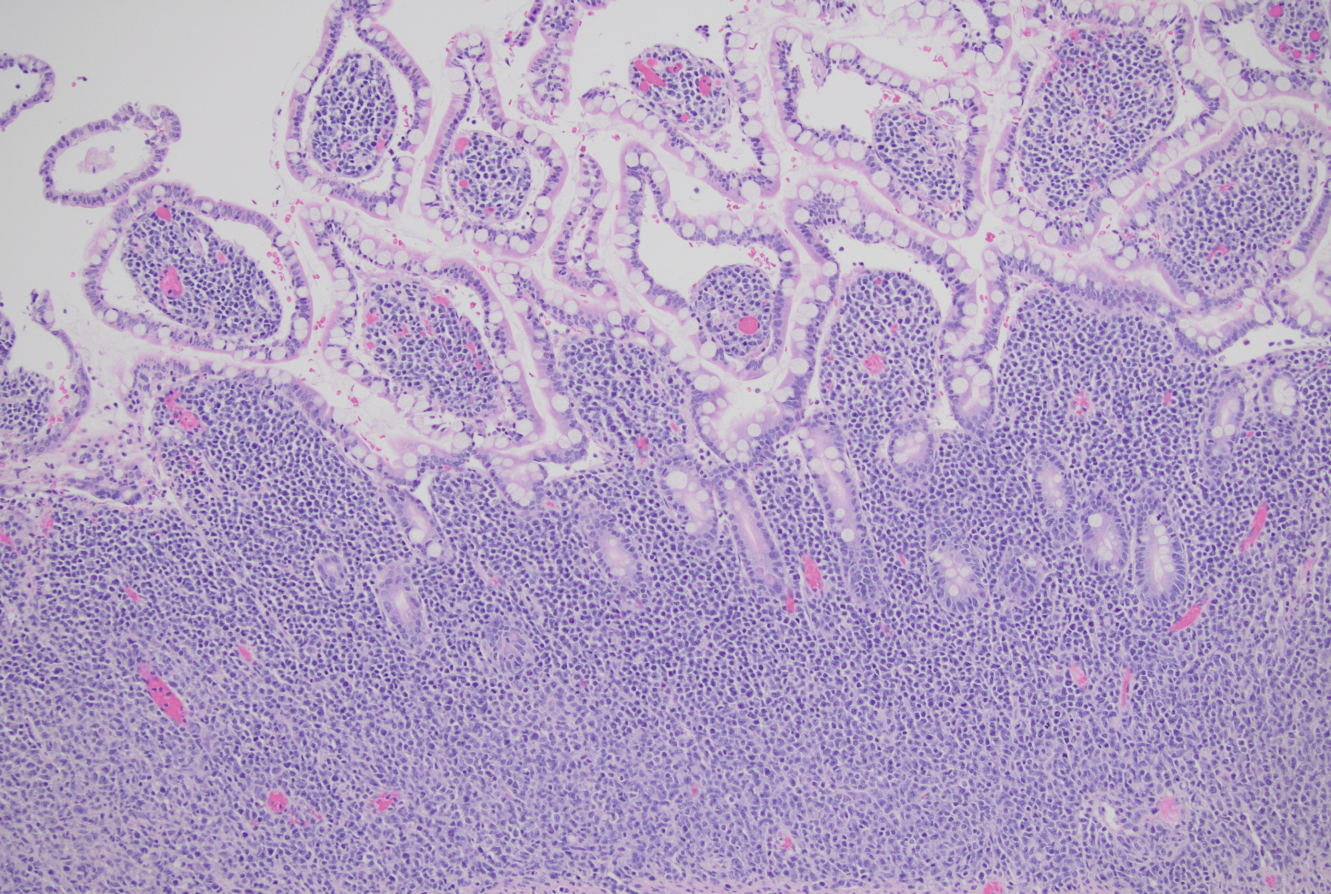
H&E stain of small bowel taken from 10/1/2024 showing diffuse large cell lymphoma within the intestinal mucosa.

Histopathologic examination of resected specimens demonstrated diffuse large B-cell lymphoma (DLBCL) with lymphoid infiltrates (Figure 5) extending from the lamina propria to the subserosa. Neoplastic cells showed irregular nuclear contours, vesicular chromatin, prominent nucleoli, and scant pale cytoplasm. Immunohistochemistry revealed CD20 positivity with co-expression of MUM1 and BCL2, but negativity for CD10, BCL6, and CD5. EBV polymerase chain reaction (PCR) showed a viral load of 65,051 IU/mL, confirming EBV-associated PTLD.

Tacrolimus and MMF were discontinued, and rituximab therapy was initiated.

The hospital course was complicated by progressive hemodynamic instability requiring norepinephrine and vasopressin, acute renal failure requiring continuous veno-venous hemofiltration (CVVH), and respiratory failure requiring mechanical ventilation following the second laparotomy. Despite aggressive surgical and medical management, he remained in shock with ongoing bleeding and worsening anemia.

Following a multidisciplinary discussion and family meeting in October 2024, care was transitioned to comfort measures. The patient died shortly thereafter due to multiorgan failure. 

## Discussion

PTLD is an uncommon but serious complication of solid organ transplantation, with incidence rates ranging from 1-3% in kidney recipients to over 10% in heart-lung recipients [1-3]. This case demonstrates an unusually aggressive presentation of GI PTLD, manifesting as massive bleeding and ischemic bowel disease shortly after renal transplantation.

Although extranodal involvement is frequent in PTLD, clinically significant GI disease is less common, accounting for approximately 10-15% of cases [4]. Prior reports describe GI PTLD presenting with recurrent bleeding and ulceration within the first year post-transplant, frequently associated with EBV infection [5-8]. Our patient’s course was consistent with these observations in terms of timing, EBV positivity, and aggressive progression; however, the degree of bowel ischemia and rapid multiorgan decline was more severe than has typically been described.

The majority of PTLD cases are EBV-associated, especially in early-onset presentations [9,10]. EBV-driven B-cell proliferation is promoted by chronic immunosuppression, particularly with calcineurin inhibitors and mycophenolate mofetil, which impair T-cell immune surveillance [9,11]. While rituximab monotherapy is well established as first-line therapy, its efficacy diminishes in advanced cases with high tumor burden or extensive GI involvement [4,11]. Surgical intervention is often necessary for bleeding or perforation, but outcomes remain poor, with mortality exceeding 50% in complicated presentations [4,7,8].

Our patient’s presentation mirrors cases described by Bawane et al. [9] and Azab et al. [11], where early EBV-positive PTLD caused severe GI hemorrhage requiring operative management. However, unlike some reports where remission was achieved with surgery plus immunochemotherapy, our patient’s course was complicated by recurrent bleeding, progressive renal failure, septic shock physiology, and ultimately multiorgan failure.

The pathogenesis of the extensive bowel necrosis in this case is likely multifactorial. Tumor infiltration, ischemia following angioembolization, and vasopressor-induced hypoperfusion may all have contributed. The profound hemoglobin decline, requirement for multiple transfusions, and fragile postoperative state further compromised intestinal perfusion. In addition, sepsis may have played a role in accelerating clinical deterioration. These factors highlight the importance of considering not only the direct oncologic burden of PTLD but also treatment-related complications and host vulnerabilities in determining outcomes.

This case highlights the critical importance of early diagnosis. EBV viral load surveillance in high-risk transplant recipients has been shown to enable pre-emptive therapy, potentially improving survival [6,9]. Emerging treatments such as EBV-specific cytotoxic T-cell infusions and checkpoint inhibitors may hold promise for refractory or rapidly progressive disease, but further research is needed [4,11].

## Conclusions

This case illustrates the aggressive nature and rapid progression of EBV-associated PTLD in the gastrointestinal tract following renal transplantation. Despite timely reduction of immunosuppression, surgical resections, and rituximab therapy, the patient experienced persistent bleeding, hemodynamic instability, and multiorgan failure. The presence of EBV-positive diffuse large B-cell lymphoma emphasizes the need for heightened suspicion of PTLD in transplant recipients with GI bleeding. Early recognition and intervention are essential, but this case highlights the significant morbidity and mortality associated with severe GI involvement. Further research is required to clarify mechanisms of treatment failure and to optimize management strategies for high-risk transplant recipients.
